# Disentangling the biopsychosocial effects of gender-affirming hormone therapy on social health: A protocol for a multi-arm prospective cohort study (AFFIRM Relationships)

**DOI:** 10.1016/j.cpnec.2025.100329

**Published:** 2025-11-26

**Authors:** Margot W.L. Morssinkhof, Nessa Millet, Giulia T. Zoppolat, Sigsten K. Stieglitz, Baudewijntje P.C. Kreukels, David Matthew Doyle

**Affiliations:** a Department of Medical Psychology, Amsterdam UMC, the Netherlands; b Center of Expertise on Gender Dysphoria, Amsterdam UMC, the Netherlands; c Amsterdam Public Health, Amsterdam, the Netherlands

**Keywords:** Social health, Trans and gender diverse, Gender-affirming hormone therapy, Voice training, Mastectomy, Protocol

## Abstract

**Introduction:**

Gender-affirming hormone therapy (GAHT) is associated with improvements in gender congruence and changes in psychosocial functioning, yet its effects on social health are not yet known. Social health, i.e., someone having adequate quantity and quality of relationships to meet their needs for meaningful connection, is a key determinant of quality of life. Understanding potential changes in social health during GAHT is therefore essential to information provision for trans and gender diverse (TGD) people. The study, *AFFIRM Relationships*, aims to prospectively examine how GAHT affects social health and to isolate the biological effects of hormonal intervention relative to other gender-affirming treatments (i.e., mastectomy and voice training).

**Methods:**

We will conduct a multi-arm prospective longitudinal cohort study of TGD people who start GAHT, gender-affirming voice training, or gender-affirming mastectomy, prospectively following participants from before starting treatment to 3, 6, 12, and 24 months after starting treatment. We will examine changes in social health, including potential changes in social networks. We aim to disentangle the ways in which social health changes after GAHT by examining changes in psychosocial functioning and the potential roles of social stigma and gender congruence. Furthermore, we will compare the effects of GAHT, which induces a systemic biological change, to the effects of voice training and mastectomy, which are non-systemic interventions, to better understand the unique biological effects of GAHT.

**Ethics and dissemination:**

Ethical approval for this study was granted by the Medical Ethical Committee of Amsterdam UMC (study no. 2024.0927). Results from this study will be disseminated via academic peer-reviewed publications, adapted into guidelines for clinical care, and we will co-design dissemination strategies for the TGD community together with a group of lived experienced experts (LEEs).

**Registration:**

N/A.

## Highlights

• Social health is understudied in trans and gender diverse (TGD) people.
• We will study how gender-affirming hormone therapy (GAHT) affects social health.
• Data are collected at 5 timepoints over 2 years in a multi-arm cohort design.
• We compare GAHT to voice training and mastectomy to isolate biological effects.
• Results will inform care and be shared with the trans community and clinicians.

## Graphical abstract

**Figure:**
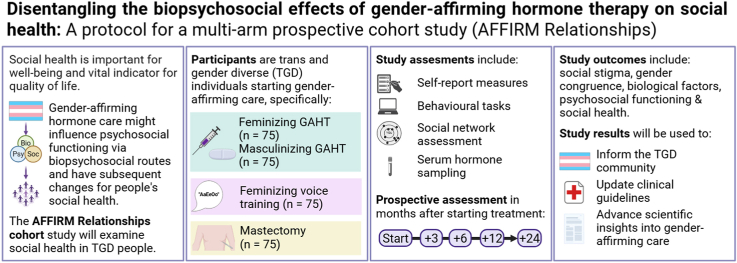

## 1 Introduction

Trans and gender diverse (TGD) people are those whose gender identity and/or expression does not align with the sex they were assigned at birth. There are several forms of medical treatment available for TGD people, and gender-affirming hormone therapy (GAHT) is commonly pursued by TGD people to better align their physical presentation with their gender [1,2]. GAHT induces biological as well as psychological and social changes (with interconnections between each of these facets). Therefore, to fully understand the impact of GAHT on quality of life, it is essential to adopt a holistic approach in examining these factors. The biopsychosocial model elaborates each of these pathways and helps healthcare practitioners (HCPs) to understand the comprehensive and diverse needs of TGD people starting GAHT [3,4]. Most past research has focused on implications of GAHT for physical and mental health, but there is a paucity of work on potential effects on social health (or interconnections between these facets), for which TGD people have highlighted a need in terms of research and clinical information provision [5].

Social health is defined as “*adequate quantity and quality of relationships in a particular context to meet an individual's need for meaningful human connection”* [6], and is a critical but understudied domain within healthcare more broadly [7]. According to the tripartite model of health proposed by Doyle & Link [6], social health sits alongside, and is interconnected with, physical and mental health, all situated within the human body and its biological processes. When social health is infrequently accounted for in healthcare research, it is generally framed as a predictor of either physical or mental health outcomes rather than as an outcome in its own right.

There is evidence of existing disparities in social health for TGD compared to cisgender people across the life course, including greater levels of family dysfunction [8], loneliness (e.g. Ref. [9]), and romantic relationship difficulties [10]. Unfortunately, research on TGD people's social networks (e.g., network size and density, relationship characteristics) and effects of gender-affirming care on social health is still scarce. Strong and supportive social relationships do lead to greater likelihood of accessing care and better outcomes of care for the individual [11], but medical interventions can also have social consequences [6]. GAHT has systemic effects on the body, including effects on the brain [12], and TGD people who start GAHT show changes in emotionality and self-regulation [[13], [14], [15]], which could impact social relationships.

Therefore, a primary way in which GAHT may affect social health is through its influence on psychosocial functioning. Although this term is used in diverse ways across the literature, here we use psychosocial functioning to refer to self- (e.g., self-control), interpersonal- (e.g., interpersonal trust) and emotional-functioning (e.g., emotion regulation). In preparation for this project, we systematically reviewed literature pertaining to changes in psychosocial functioning after GAHT, identifying 46 studies [13]. Our review highlighted relatively consistent evidence for reductions in psychological distress and depressive symptoms after GAHT, but more mixed and inconclusive findings for other outcomes, such as general and social anxiety symptoms, emotion expression, and self-control. These studies did not consistently or regularly account for important potential confounders (e.g., body image, sexuality) or incorporate control groups to bolster causal inference. Moreover, the temporal nature of changes in psychosocial functioning, including how they may fluctuate or shift from one timepoint to another, has not been adequately considered.

To further explore potential changes, we conducted semi-structured interviews with 26 TGD people on their experiences of masculinizing and feminizing hormone therapies (Millet et al., in prep). Key findings suggested that access to GAHT and noticing its physical effects provided a sense of safety for participants. This, in combination with reduced gender dysphoria, was instrumental in participants gaining self-confidence and courage to engage socially and to feel agentic in decision-making around relationships and social networks. Feminizing and masculinizing GAHT were seen to affect participants' mood, self-regulation, and emotionality in distinct ways, which positively and negatively influenced participants' responses to conflict and ability to connect with others.

**Fig. 1:**
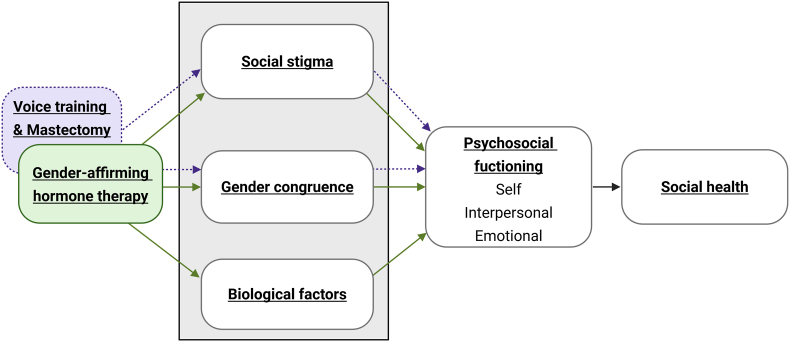
Theoretical model underlying AFFIRM Relationships. The colored boxes on the left display the gender-affirming medical treatments in the study, the “black box” contains the possible mechanistic routes through which these treatments could affect psychosocial functioning and, thereby, social health. Social stigma includes social gender affirmation and minority stress, gender congruence includes gender congruence and euphoria, biological factors include changes in hormone levels.

Collectively, these studies indicate multiple potential pathways by which GAHT can affect psychosocial functioning. However, as reviewed in Doyle et al. [13], quantitative studies thus far have mostly examined psychosocial functioning changes after GAHT using a “black box” approach, viewing GAHT as the input and psychosocial functioning as the output, without any indication of which underlying biopsychosocial mechanisms are contributing to these effects. Given that there are most likely paths from GAHT to psychosocial functioning through social stigma (e.g., via passing in society) and gender congruence (e.g., via reduced dysphoria) in addition to direct biological factors (e.g., changes in hormone levels and subsequent physiological and neurobiological changes), this is an essential oversight in research thus far. In our study, we therefore aim to conduct a theory-informed analysis of biopsychosocial factors sitting within the “black box” by assessing social stigma, gender congruence, and biological factors which are affected by GAHT in order to disentangle effects via these distinct routes. The theoretical model underlying our study, which connects GAHT to changes in social health via such pathways, is shown in Fig. 1.

With this study, we aim to better inform TGD people, their close others, and HCPs about the possible psychosocial effects of GAHT, so that they can account for what changes they might expect in their (social) lives, including the structure of their social network, after starting GAHT.

## 2 Methods and analysis

### 2.1 Study setup

The overall aim of *AFFIRM Relationships* is to use a prospective research design to examine changes in social health among TGD people after starting GAHT from a biopsychosocial perspective. Below, we outline the different research questions and hypotheses of the *AFFIRM Relationships* study.

#### 2.1.1 Research question 1: what are the effects of GAHT on social health, and are changes in social health mediated by changes in psychosocial functioning?

First, we are interested in how social health is influenced by GAHT, and whether changes in psychosocial functioning underpin the association between GAHT and social health over time (before the start of GAHT, at 3 months, 6 months, 12 months, and 24 months following the start of GAHT).

We hypothesize that social health will improve after starting GAHT, and this change will be mediated through general improvement in psychosocial functioning. However, we also expect that some aspects of psychosocial functioning (e.g., emotion regulation, emotion recognition) may be impaired, and we expect differing effects dependent on GAHT type (i.e., masculinizing vs. feminizing).

#### 2.1.2 Research question 2: which effects of gender-affirming treatment on psychosocial functioning and social health are unique to the GAHT group, possibly due to biological systemic effects of hormones?

Second, we aim to differentiate the effects of GAHT from other forms of gender-affirming care (without systemic biological effects) in order to best isolate a potential biological pathway through which GAHT affects psychosocial functioning. We do so by including TGD people starting gender-affirming voice training or undergoing a gender-affirming mastectomy as comparison groups, allowing an assessment of the differences between non-hormonal and hormonal forms of gender-affirming treatment to disentangle effects of biological factors, social stigma, and gender congruence on psychosocial functioning and consequently social health outcomes.

We hypothesize that there will be differences and similarities in the effects of hormonal and non-hormonal gender-affirming treatments on psychosocial outcomes and these will be explained by differences/similarities in social stigma, gender congruence, and biological pathways.

#### 2.1.3 Research question 3: how do social networks change after starting gender-affirming treatment, and do these changes influence outcomes of gender-affirming care?

Third, we aim to assess the dynamic relationships between social networks and gender-affirming care. We will investigate changes in social networks after starting gender-affirming treatment, including structural changes (e.g., number of people in social network, reported closeness to others, ties between social network members) as well as functional changes (e.g., relationship quality, perceived support of gender and transition), as well as potential effects of networks on care-related outcomes.

We hypothesize that after starting gender-affirming treatment, participants will report changes in social networks in line with improved social health, possibly reporting, for example, decreases in low-quality contacts (e.g., unsatisfying or unaffirming relationships) and increases in high-quality contacts (e.g., satisfying and affirming relationships), an increase in the proportion of social network members who are also TGD (or members of the LGBTQ + community) and/or who show higher rates of support for one's gender and transition.

### 2.2 Study design

Investigation into GAHT has thus far heavily relied on the use of cross-sectional designs, which come with significant limitations if one aims to draw causal inferences. Since randomized controlled trials (RCTs) on GAHT have limited clinical generalizability and pose significant ethical and practical challenges [16], we will use a longitudinal within-person design, in which we measure changes in psychosocial functioning and social health in TGD people starting gender-affirming treatment. Within this longitudinal cohort design, we therefore organize the study into three arms (including two comparison arms): one arm of participants starting feminizing or masculinizing GAHT, one arm of participants starting feminizing voice training, and one arm of participants undergoing a masculinizing mastectomy (i.e., masculinizing top surgery). All three forms of gender-affirming care have significant effects on gender expression and congruence [13,[17], [18], [19]], whereas GAHT is the only form of treatment to have systemic biological effects. This multi-arm group design therefore enables us to better disentangle the possible biological effects of GAHT from the social stigma- and gender congruence-related effects also seen in other forms of gender-affirming care. Altogether, the prospective within-person assessments will equip us with more power to point to possible causal pathways in changes in psychosocial functioning after starting GAHT. These comparison conditions can also provide better insights into the effects of voice training and mastectomy surgeries, contributing directly to evidence-based care for TGD people. A visualization of the study's theoretical model and how the various treatment groups are integrated is shown in Fig. 1. Thus, this is a multi-arm observational cohort study which follows TGD people who start gender-affirming treatment (GAHT, gender-affirming voice training, gender-affirming mastectomy) in the Netherlands over 24 months.

### 2.3 Community involvement

TGD people with lived experience of GAHT and their close others, referred to as Lived Experience Experts (LEEs), have been engaged throughout the research trajectory of this ongoing project. LEEs are engaged in sessions with the aim of: co-creating and defining research priorities, consulting on study materials and methods, sense checking findings, and co-producing dissemination strategies [14]. During conceptualization and study design phases of this project, LEEs were engaged in a co-creation session to explore their experiences of changes in social health and relationships when starting GAHT. We used the Equitable and Power Sharing Approach (EPSA) method [14] to promote reflexivity on the part of the research team, create a safe environment for LEEs to engage in open dialogue, and enable LEEs to partake in scientific research activities. Using the EPSA method, insights derived from co-creation were then combined with theoretically informed knowledge to produce a refined conceptual model for the current cohort trial (shown in Fig. 1).

### 2.4 Our approach as a research team

We conceptualise this study from the multiple perspectives of a research team from varying academic backgrounds, including social and health psychology, public health, psychiatry, and neuroendocrinology. While most team members have ample experience of conducting research in the context of transgender healthcare within hospital settings, no members of the team work in a clinical capacity. Our approach is guided by the following core principles: a commitment to producing research which will better enable TGD people to inform themselves and therefore enable informed decision-making about their medical care; utilization of a biopsychosocial perspective which positions social health as a key outcome of medical intervention and considers this alongside mental and physical health; and dedication to using a participatory research framework which integrates knowledge derived from community insights alongside scientific theory and empirical findings [14]. We want to acknowledge that changes after starting GAHT are embedded in everyday lives, which are complex, dynamic and nuanced. This means that exclusively studying “clinical outcomes” most likely will not capture a holistic picture of the effects of GAHT on quality of life. Therefore, we also focus on studying “non-clinical outcomes” of care (e.g., social networks), and over time, consciously moving towards a more nuanced picture of GAHT's effects.

### 2.5 Setting

This study is embedded in the Centre of Expertise on Gender Dysphoria (CEGD) at Amsterdam UMC in the Netherlands. The CEGD offers multidisciplinary care for TGD people of all ages, admitting approximately 400 adults and 200 adolescents every year. Participants will be recruited via the CEGD and external clinical partners providing similarly structured gender-affirming treatment as necessary to reach recruitment targets.

### 2.6 Participants

#### 2.6.1 Eligibility criteria

Only those who are 18 years of age or older, who speak and write proficiently in English and/or Dutch, and who are hormone naïve (i.e., have not previously used GAHT) will be considered for inclusion. Those with significant psychological comorbidities making it likely that study participation will be overly burdensome will be ineligible. For all groups, this will be determined by the person's HCP.

#### 2.6.2 GAHT group

We will approach TGD people who are due to start GAHT in a Dutch clinical setting. Specifically, TGD people will be approached when they are either nearing the end of the “diagnostic” phase or when they have received approval to start GAHT, but have not yet started GAHT.

#### 2.6.3 Active comparison groups: voice training group & mastectomy group

For the voice training group, we will approach TGD people who are due to start gender-affirming voice training and who have never had gender-affirming voice training before. For the mastectomy group, we will approach those who are due to undergo a mastectomy at participating centres via their respective HCPs. Participants in the voice training group and mastectomy group should also be hormone naïve, meaning they have never used gender-affirming hormones.

### 2.7 Gender-affirming treatment protocols

#### 2.7.1 Gender-affirming hormone therapy

Participants in the GAHT group of the study will start GAHT as a part of a medical care trajectory in the Netherlands, and the hormone treatment will be medically supervised by a trained medical doctor throughout the study. All participants in the GAHT group will have regular appointments with a mental health care professional during the first 12 months of GAHT, as part of standard clinical care. Masculinizing GAHT will include the administration of testosterone and optional use of menstrual cycle suppressants (i.e., progestins or hormonal contraceptives). Feminizing GAHT will most commonly consist of estradiol combined with anti-androgens. We want to emphasize that there are continuous innovations and an increasing diversity in GAHT options, including non-use of anti-androgens, lower dosages of hormones, additional use of progesterone, or temporary use of hormones, and that the specific forms of GAHT used could therefore change during data collection for this study. Participants’ treatment will be in line with care guidelines as recommended by professional organizations, including the World Professional Association of Transgender Health (WPATH) standards of care [1] and the clinical practice guidelines of the Endocrine Society [20]. Hormone levels will be measured as part of the standard treatment protocol, as recommended by the WPATH standards of care, and hormone treatments will be adjusted to optimize desired treatment effects and safety.

#### 2.7.2 Voice training

Participants in the voice training group will receive voice training from speech therapists in the Netherlands who followed formal training in providing gender-affirming voice training and are therefore certified to give gender-affirming voice training. Additionally, providers need to have at least 1 year of experience providing this type of care. Voice training will comprise several training techniques, including but not limited to pitch elevation training and articulation-resonance training, which have been proven to be effective in changing the voice to sound more feminine [21]. Voice training is focused on voice changes, and psychotherapy (e.g., treatment for social anxiety) is not part of voice training in this study. If participants in this group start GAHT during the study, they will be offered the opportunity to be re-included in the GAHT group (although prior data provided by these participants will still be included in the analyses of the voice training group).

#### 2.7.3 Mastectomy

Participants in the mastectomy group will undergo a mastectomy which will be performed by a surgeon trained to provide gender-affirming mastectomies in the Netherlands. Participants may undergo one of the following mastectomy surgical techniques, including keyhole technique, donut procedure, batwing procedure, double incision, or inverted T. Additionally, they may undergo revision surgeries after the mastectomy if deemed necessary. Similarly to the voice training group, if participants from this group start GAHT, they will be offered the opportunity to be re-included in the GAHT group (although prior data provided by these participants will once again still be included in the analyses of the mastectomy group).

### 2.8 Recruitment

Eligible participants will be approached by their HCPs, who will inquire whether the client is interested in study participation and then either ask consent from the client to be contacted by the research team, or alternatively, will point the client toward the custom-built secure contact form via which they can use to directly contact the research team. A member of the research team will then contact the client to provide more information about the study and screen for remaining eligibility criteria. Those still interested in participation will receive the full study information sheet and a virtual appointment will be scheduled to answer any remaining questions and to complete informed consent.

### 2.9 Study participation logistics

Participants in each group will complete an online questionnaire at baseline (i.e., before starting their form of gender-affirming treatment) and at 3, 6, 12, and 24 months after starting their respective gender-affirming treatment. For the GAHT and mastectomy groups, the baseline measurement will take place after the participant receives approval to get GAHT or a mastectomy. Participants in the GAHT group will participate in behavioral measurements at study visits on the day of initiating hormones and after 3 and 12 months of GAHT (Fig. 3).

#### 2.9.1 Questionnaires

Participants will be invited to complete study questionnaires via a scheduled invitation email, and the questionnaires will all be completed online using the platform Castor EDC. The study questionnaires are estimated to take 70 min at baseline and 12 months of gender-affirming care, and 40 min at 3, 6, and 24 months of gender-affirming care.

#### 2.9.2 Study visits

The GAHT group will additionally attend study visits which are planned on the same day as routine clinic visits. During these study visits, they will complete a *personal social network task* in a custom-built user interface in Network Canvas [22]. Furthermore, participants will complete behavioral tasks focused on examining *implicit gender attitudes*, *emotional inhibition*, *interpersonal trust*, and *self-control*. In both the behavioral measurements and the collection of the social network data, participants will complete the tasks independently but a research team member will be available for questions. Each study visit is expected to take around 60 min.

#### 2.9.3 Video meeting for social network assessment

An online video call will be scheduled with participants in the mastectomy or voice training groups in which they will complete the *social network task* in the same week as their questionnaire completion. As with the GAHT group, participants will complete the tasks independently but will be able to ask questions to the research team member if needed. Each video meeting with the research team is estimated to take around 20 min.

### 2.10 Measures

See Supplementary Table 1 for a full list of all study constructs, with corresponding instruments and references, and Fig. 4 for the placement of these constructs within the proposed conceptual model. We selected the questionnaires and instruments based on construct alignment (i.e., alignment with the construct we want to measure), face validity of the items, and instrument validation (i.e., has the questionnaire been previously validated?). The full questionnaires for each group and time point can also be found on the study OSF page (osf.io/zjkhv/).

#### 2.10.1 Demographics and covariates

Participants will complete a series of demographics and potential covariates. At baseline and several follow-up timepoints, we will assess characteristics which enable us to conduct intersectional analyses, including but not limited to *age, gender, sexual and romantic orientation, socioeconomic status, migration background and ethnicity, education, mental healthcare, autism symptoms,* and *autism diagnosis status.*

#### 2.10.2 Social stigma

To measure social stigma, we will use three measures assessing factors related to social stigma (and resilience), including *social gender affirmation*, *TGD minority stress*, and *sexual objectification*. In selecting these outcomes, we opted to include both positive (e.g., gender affirmation) and negative (e.g., minority stress) factors, as well as relevant constructs proposed by the LEE group (e.g., sexual objectification; [14]).

#### 2.10.3 Gender congruence

We opt to use four measures assessing different aspects of gender congruence, including *gender congruence*, *gender euphoria*, *appearance satisfaction*, and *body appreciation*. Additionally, participants in the mastectomy control group will also complete a measure of *chest and nipple satisfaction,* as well as measures assessing *body expectations* at baseline and *effects of body image on life* at every timepoint. Participants in the voice training group will complete a measure of *voice satisfaction*.

#### 2.10.4 Biological factors

To examine possible biological factors in the GAHT group, we will collect *serum hormone levels*, which are measured as part of routine clinical practice, in the GAHT group; we will ask participants about their *form and dosage of GAHT* to determine possible dosage- or administration-dependent effects of GAHT.

#### 2.10.5 Psychosocial functioning

To measure psychosocial functioning, we opt to use measures assessing three facets of psychosocial functioning: self-, interpersonal-, and emotional-functioning. Participants in the GAHT group will additionally complete behavioral measures assessing *implicit gender associations*, *emotional inhibition, interpersonal trust,* and *self-control*, which will enable us to triangulate questionnaire data with behavioral tasks for more robust conclusions.

#### 2.10.6 Social health

We will examine social health using both global measures of social health and nuanced evaluation of personal social networks. We will assess participant's *social networks* using a sociogram, as illustrated in Fig. 5, which captures the social connections between one person (the “ego”) and their social contacts (“alters”), as well as the connections between alters in the network. Best practices and recommendations for eliciting high quality network data (e.g., Ref. [23,24]) were followed in design and measurement decisions. Participants will first be asked to nominate up to 20 people that are in their social network. They will then be shown three concentric circles and asked to imagine themselves at the middle of the circles and to place each alter in the circle based on their closeness to the self and how close the alters are to each other. Participants will then complete measures assessing alter properties (e.g., *age, gender, relationship type, interaction frequency*), ego-alter properties (e.g., *support of transition, relationship satisfaction, conflict*), and alter-alter properties (i.e., *presence and quality of relationship*).

### 2.11 Statistical analyses

#### 2.11.1 Planned analyses

To address RQ1, i.e., effects of GAHT on social health and the mediating role of psychosocial functioning, we will conduct a longitudinal mediation analysis using multilevel models in which we examine the effects of GAHT on social health and the possible mediating role of self-, emotional-, or interpersonal-functioning on this association. In this analysis, we will only include participants who started GAHT. This analysis is theory-informed and tests our a priori hypothesis, which is that social health improves after starting GAHT due to changes in aspects of psychosocial functioning. The power analysis and sample size estimate for the study were based on this first RQ1 analysis. To complement these hypothesis-driven primary analyses, we will conduct additional analyses in which we examine associations between different aspects of psychosocial functioning and social health, and in which we also stratify analyses by GAHT types (i.e., masculinizing and feminizing GAHT).

**Fig. 2:**
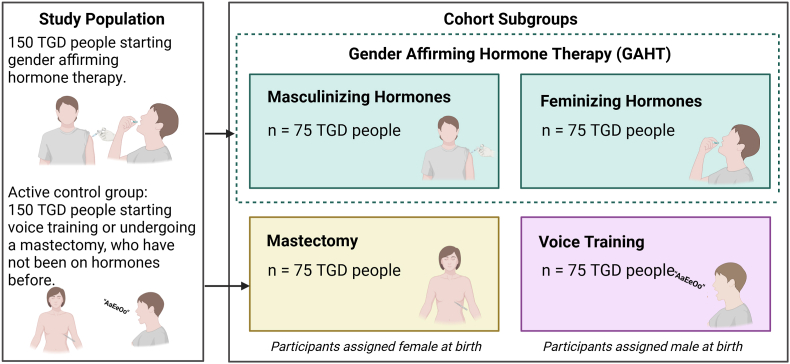
Description of study participants and participant grouping. Note that participants in the mastectomy and voice training groups can move into the GAHT group if applicable (i.e., if they start GAHT during the course of participation in the study).

To answer RQ2, i.e., differences between a biological, systemic gender-affirming treatment (i.e., GAHT) and non-systemic gender-affirming treatments (i.e., mastectomies and voice training), we will further examine changes in the aforementioned “black box”, i.e., the connection between gender-affirming treatment and changes in psychosocial functioning. To do so, we will conduct a second longitudinal mediation analysis using data from all groups, analyzing changes in the different facets of psychosocial functioning after participants start gender-affirming treatment and the possible mediating roles of social stigma, gender congruence, and biological factors. In this analysis, we will also account for the type of gender-affirming treatment as visualized in Fig. 2, meaning this analysis will offer group-level insights into the different effects of GAHT, voice training, and mastectomy.

**Fig. 3:**
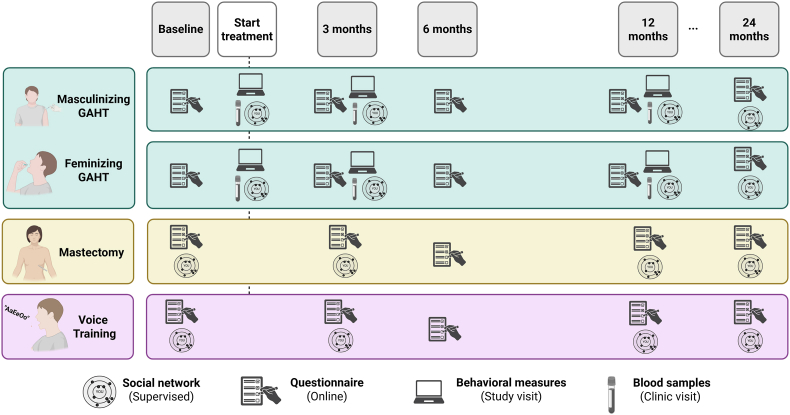
Timeline of tasks and study visits for each participant group. *Note*: *Behavioral measures will be completed at in-person study visits for the GAHT group at baseline,* 3 months*, and* 12 months *of GAHT, and blood samples are measured at baseline, 3, and* 12 months *of GAHT as part of ongoing clinical care.*

**Fig. 4:**
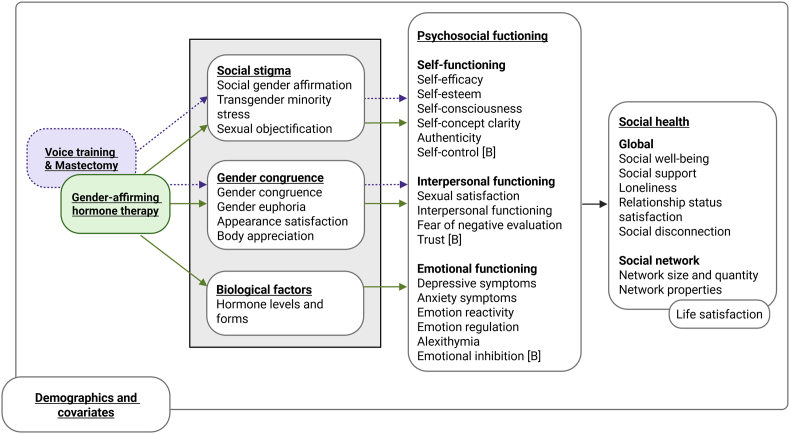
Placement of study measures in the theoretical model. All constructs are self-reported, with the exception of constructs with a [B] noted next to them, which are measured using behavioral tasks.

**Fig. 5:**
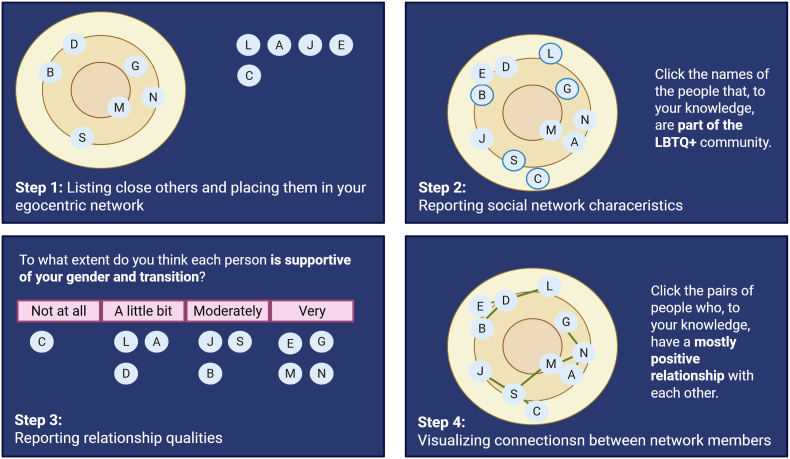
Example of personal social network assessment and related questions, as administered in Network Canvas.

To answer RQ 3, i.e., changes in social networks after starting gender-affirming treatments, we will examine various network properties at baseline and over the course of transition (e.g., number or proportion of supportive others compared to unsupportive others; the presence of TGD people in the network; the density of the network), as well as how baseline network characteristics (or changes in these) might predict treatment-related outcomes (e.g., life satisfaction). Further, we will explore whether there are differences in these network properties between the treatment groups.

#### 2.11.2 Secondary analyses

For analyses outside of the primary analyses articulated above, we will formulate research analysis plans and preregister these wherever possible. These analyses could be informed by those with lived experience, professional capacity, or new research insights.

### 2.12 Sample size considerations

We base our sample size considerations on the latest sample size recommendations for the primary research questions of the project, as well as attrition considerations based on previous research and feasibility and budgetary constraints [25]. The main analyses planned in the project are focused on multilevel longitudinal mediation, examining whether psychosocial functioning plays a mediating role in changes in social health in the 2 years after starting GAHT.

Assuming that GAHT has at least a small-to-medium-sized effect on psychosocial functioning (Cohen's *d* of 0.26; [26,27]) and psychosocial functioning has a medium-sized effect on social health (Cohen's *d* of 0.39; [28]), a sample of 100 participants at 5 measurement time points would result in 0.8 power in longitudinal mediation analyses [29], assuming a relatively high *ICC* of 0.6 for psychosocial functioning [30].

We will assume a loss to follow-up of about 30 % (e.g. Ref. [31]). We therefore aim to include a sample of 150 participants starting GAHT and 150 participants starting non-hormonal gender-affirming treatment (see Fig. 2). For the active comparison groups, we matched the sample sizes to the participants with the same sex assigned at birth (i.e., 75 participants undergoing a mastectomy and 75 participants starting voice training). This will provide sufficient power (.80) to conduct mediation analyses in the GAHT group.

We aim to minimize loss to follow-up in multiple ways in the study setup. First, we have designed the study with participation from LEEs, enabling us to include outcome measures which are also of interest to the TGD community, and will act as a motivation for sustained participation. Second, we have ensured that participants receive adequate reimbursement for the time they spend on study participation (i.e., reimbursement determined by institutional guidelines, which recommend time-based reimbursements set on the national minimum wage). Third, we will prioritize direct participant contact throughout the study, ensuring that the study team is easy to reach, participants’ questions are answered, and that the study setup is clear so that study participation is as easy as possible for participants.

### 2.13 Ethical approval & data management

Ethical approval was granted by the Medical Ethical Committee of Amsterdam UMC (no. 2024.0927). Any significant changes to the study protocol will be subject to a protocol amendment and these changes will be tracked in the study materials posted on OSF (osf.io/zjkhv/). This study has been approved as non-WMO, which means that it does not fall under the Dutch law for medical scientific research with humans.

### 2.14 Open science

In highly polarized fields of research, including research on gender-affirming treatment, it is vital to build trust among relevant stakeholders and the public through transparency and responsibility in the conduct and reporting of research. To do this, we embrace open science practices and FAIR (Findable, Accessible, Interoperable, Re-useable) data principles, along with centering community involvement via transparent and active engagement throughout the research process. At the same time, it is necessary to recognize that research on politicized topics involving members of marginalized groups (e.g., TGD people) requires careful attention to issues of confidentiality and sensitivity. Therefore, consistent with EU guidelines [32], we will make our work "as open as possible, as closed as necessary." In addition to this protocol, further analyses of data from the *AFFIRM Relationships* cohort will be preregistered on the Open Science Framework (osf.io). All data analysis scripts will be uploaded subsequent to publication of academic papers. Due to issues of identifiability and vulnerability of the participant population, only summary data will be made available on OSF. Parties or collaborators interested in using the study data are invited to write a paper proposal, outlining the analysis aim and method, and upon approval of the paper proposal they will be granted access to the relevant data in a secure workspace (i.e., MyDre environment hosted by Amsterdam UMC). Preprints of academic papers along with open access copies of final articles will also be uploaded to OSF.

### 2.15 Dissemination

There will be 3 research dissemination streams to share the results of this study, which are oriented toward 1. academia, 2. policy, and 3. community.

1. We will disseminate findings via traditional academic routes such as sharing findings on the project website (affirmrelationships.com), via abstract and oral presentation at domain-specific and more general academic conferences, and publication in scientific peer-reviewed journals and publication to the general public via mass media (e.g., social media such as Instagram and BlueSky, newspapers articles).
2. We will consult with healthcare practitioners, policy-makers, and researchers working in the field of transgender health to use the findings of this study to update standards of care and best practice guidance for GAHT (and other gender-affirming treatment).
3. We will co-design dissemination strategies with the project's LEE members to share the study findings within the TGD community. Design of these strategies will be guided by TGD people, with a focus on creating accessible information that can reach diverse groups of TGD individuals. Such strategies may include delivering inclusive workshops, translating the information into creative outputs, or making a podcast.

## 3 Discussion

Social health of TGD people is an understudied topic despite its central role in quality of life as well as its significance to healthcare. TGD people have voiced a need for greater information provision on how their social health may shift when starting GAHT in order to better prepare for navigating gender transition [5]. With this study, we aim to gain insights about the effects of GAHT on TGD people's social health (including their social networks), and on the mediating role of psychosocial functioning at varying time points in the first 2 years of GAHT. Furthermore, we aim to disentangle the various biological and non-biological effects of GAHT by including other TGD groups starting gender-affirming treatment (i.e., voice training and mastectomy). The conceptual model for this study has been developed in collaboration with LEEs to ensure that findings are not only theoretically relevant but also practically relevant for TGD people and their close others [14].

### 3.1 Methodological advances

Our approach advances the current state of the science on GAHT in various ways. We opt to include active control groups, which enables direct comparison of the assumed biological effects of GAHT to shared non-biological effects (e.g., via social stigma and gender congruence) of other forms of gender-affirming treatment, making the study quasi-experimental [33]. This choice, as well as the longitudinal set-up, bolsters our ability to infer causality from our findings while simultaneously providing further insights into the effects of different forms of gender-affirming treatment [34]. In addition, we will examine the possible mechanisms by which GAHT could affect psychosocial functioning and then social health, instead of using the “black box” approach, which does not address potential underlying mechanisms. We also opt to use measures of psychosocial functioning that are embedded in everyday life (e.g., emotion reactivity, interpersonal trust) in lieu of strictly clinical outcomes [5,35]. These measures could be more sensitive to changes in the daily lives of TGD people using GAHT and could therefore better reflect the pathways which possibly contribute to changes in social health. The addition of behavioral measures examining the three core aspects of psychosocial functioning (self-, emotional-, and interpersonal-functioning) enables triangulation with self-report measures to provide more in-depth insights. Such triangulation results in increased nuance of interpretation and a clearer picture of how GAHT influences social health, which can contribute relevant knowledge for TGD people and their close others.

This study is further bolstered by using a participatory research approach to healthcare as a guiding framework. A participatory research approach ensures that study processes are designed so that TGD people and their close others can play an active role in guiding research priorities [36]. This is particularly relevant when researching GAHT, a medical treatment that strongly influences the well-being of TGD people and in the appraisal of which TGD people have a significant “stake” [5]. Thus, we are committed to positioning community-generated insights as legitimate knowledge at various points of this study (e.g., decision-making during study design, acceptability of research methods, and framing of results dissemination). In particular, we advance the use of participatory health research by endorsing transparency when integrating community-derived insights [14,37] alongside using methods to deconstruct power hierarchies embedded in traditional research practices [38,39]. We aim to continue embedding this study in principles of transparent engagement to promote empowerment of TGD people and enable power to be shared equitably throughout the research trajectory. Importantly, results from this study will be shared with LEEs to consider how these can be framed to serve the purposes of the research while also providing helpful information the TGD community.

### 3.2 Relevance for practice

Improved insights into the effects of GAHT are also directly relevant to HCPs working in the field of transgender health. Guidelines for treating TGD people refer to the importance of informing people in a way that enables well-informed decision-making [1,20]. Well-informed decision-making is often conceptualized as the ability to weigh risks and benefits of a treatment. However, what is perceived as a risk or a benefit may vary from person-to-person, including between TGD people themselves and their HCPs. Information that should be provided may consist of what is known regarding effectiveness and safety, possibilities and limitations of certain treatment options, short- and long-term effects of treatment, possible worsening or improvement of other conditions, and unfavorable and favorable psychological and social outcomes.

TGD people report that the information provided in clinical practice is predominantly biomedical (including the onset and time course of physical changes, possible physical health outcomes, and medical risks) and lacks narratives of TGD people's treatment experiences, including what treatment-related changes entail in daily living and in their social world [14,[40], [41], [42]]. Individuals who report being poorly informed describe that the information provision about topics such as psychosocial aspects of transitioning is insufficient [42]. In the absence of nuanced information provision by HCPs, TGD people seek this information via other channels, such as (social) media, peers, and community groups [43]. Because not everyone has similar access to such channels of information, knowledge about what people on GAHT actually experience should also be available in healthcare settings for TGD people and their close others. With this study we aim to gain insights into the effects of GAHT on social health, and to better isolate the biological effects of GAHT on psychosocial functioning and social health. These outcomes will directly improve information provision about GAHT for TGD people and their loved ones, and thereby will improve care.

## CRediT authorship contribution statement

**Margot W.L. Morssinkhof:** Writing – review & editing, Writing – original draft, Visualization, Project administration, Methodology, Formal analysis, Conceptualization. **Nessa Millet:** Writing – review & editing, Writing – original draft, Project administration, Methodology, Conceptualization. **Giulia T. Zoppolat:** Writing – review & editing, Writing – original draft, Methodology, Conceptualization. **Sigsten K. Stieglitz:** Writing – review & editing, Writing – original draft, Visualization. **Baudewijntje P.C. Kreukels:** Writing – review & editing, Writing – original draft, Supervision. **David Matthew Doyle:** Writing – review & editing, Writing – original draft, Supervision, Methodology, Funding acquisition, Formal analysis, Conceptualization.

## Funding statement

Funded by the 10.13039/501100000780European Union (ERC-StG 101042028). Views and opinions expressed are however those of the author(s) only and do not necessarily reflect those of the European Union or the European Research Council Executive Agency. Neither the 10.13039/501100000780European Union nor the granting authority can be held responsible for them. The funders had no role in the study design, collection, analysis, and interpretation of data, writing of the article, or the decision to submit for publication.

## Declaration of competing interest

The authors declare that they have no known competing financial interests or personal relationships that could have appeared to influence the work reported in this paper.
