# Supplementary material for: Disentangling the biopsychosocial effects of gender-affirming hormone therapy on social health: A protocol for a multi-arm prospective cohort study (AFFIRM Relationships)
Source: Compr Psychoneuroendocrinol. 2025 Nov 26;24:100329. doi: 10.1016/j.cpnec.2025.100329 (PMC12702236; doi:10.1016/j.cpnec.2025.100329)
Supplement: Multimedia component 1 [file mmc1.docx]

**Supplementary materials**

Supplementary Table 1 displays all measurement instruments used, including example questions and timepoints.

| **Supplementary Table 1. Variables and instruments used for main measures in the study.** | | | | | | | | | | | |
| --- | --- | --- | --- | --- | --- | --- | --- | --- | --- | --- | --- |
| **Variable** | **Instrument** | **Item example** | **Baseline** | | **3 months** | **6 months** | | | **12 months** | **24 months** | |
| **DEMOGRAPHIC** | | | | | | | | | | | |
| Age | Self-constructed | *How old are you?* | X |  | | |  | X | | | X |
| Gender | Self-constructed | *What would best describe your gender identity?* | X | X | | | X | X | | | X |
| Living situation (social context) | Self-constructed | *What is your current living situation?* | X | X | | | X | X | | | X |
| Financial strain | Household strain (Okechukwu et al., 2012) | *How would you describe the money situation in your household right now?* | X |  | | |  | X | | | X |
| Parental status | Self-constructed | *Do you have any children?* | X |  | | |  | X | | | X |
| Education and employment status | Self-constructed | *How would you describe your current work status?* | X |  | | |  |  | | |  |
| Ethnicity | Self-identification | *How would you describe your ethnicity?* | X |  | | |  |  | | |  |
| Migration background | Birthplace and parental birthplace | *Were you born in the Netherlands?* | X |  | | |  |  | | |  |
| Religious belief | Self-constructed | *Do you practice a specific religion right now?* | X |  | | |  |  | | |  |
| Sexual and romantic orientation | Gender-Inclusive Scale (Galupo et al., 2017) | *Please rate to what extent you are sexually attracted to each of the following groups…* | X |  | | |  | X | | | X |
| Subjective social status | MacArthur ladder | *Think of a ladder as representing where people stand. Please place a large X on the rung where you think you stand at this time in your life relative to other people in the Netherlands* | X |  | | |  | X | | | X |
| Relationship status, no of partners, length, living with partner, type, events, loss of relationship | Self-constructed | *Please select the answer that best describes your current relationship status.* | X | X | | | X | X | | | X |
| Gender affirmation steps | Self-constructed | *Do or did you want hair removal, and if so, have you started hair removal?* | X | X | | | X | X | | | X |
| Autism symptoms | Autism Spectrum Quotient-10  (Allison et al., 2012) | *I find it easy to read between the lines when someone is talking to me.* | X | X | | | X | X | | | X |
| Autism status | Self-constructed | *Do you have a formal diagnosis of Autism/ASD/ASS?* | X |  | | |  | X | | | X |
| Autistic masking | Masking subscale of the Camouflaging autistic traits (CAT-Q) scale (Hull et al., 2019) | *I adjust my body language or facial expressions so that I appear interested by the person I am interacting with.* | X |  | | |  | X | | | X |
| Sex drive | Modified version of the Sexual Desire Inventory (Spector et al., 1996) | *During this last month, how often have you had sexual thoughts involving a partner?* | X | X | | | X | X | | | X |
| Personality | Big Five Inventory-10 (Rammstedt & John, 2007) | *I see myself as someone who is outgoing, sociable.* | X |  | | |  |  | | |  |
| Attachment style | Experiences in Close Relationships-Relationship Structures questionnaire (Fraley et al., 2011) | *I usually discuss my problems and concerns with others.* | X |  | | |  | X | | | X |
| Desirable responding | Marlowe-Crowne scale (Crowne & Marlowe, 1960) | *On a few occasions, I have given up doing something because I thought too little of my ability.* | X |  | | |  |  | | |  |
| Gender role attitudes | Social roles questionnaire with modified answer scale (Baber & Tucker, 2006) | *People can be both aggressive and nurturing regardless of sex.* | X |  | | |  | X | | | X |
| Trans Group identification | In-group identification scale Leach and Spears (Leach et al., 2008) | *I think that trans people have a lot to be proud of.* | X | X | | | X | X | | | X |
| Experiences on hormones (exclusive of GAHT) | Self-constructed | *Did, or do, you experience any of the following side effects of being on the oral contraceptive pill?* | X |  | | |  |  | | |  |
| Premenstrual experiences | Premenstrual Symptom Screening Tool (Steiner et al., 2003) | *Do or did you experience any of the following premenstrual symptoms which start before your period and stop within the first days of bleeding?* | X |  | | |  |  | | |  |
| Persistent menstrual bleeding/spotting | Self-constructed | *Do you still have periodic or menstrual-like blood loss or spotting?* | X | X | | | X | X | | | X |
| Fear of being single | Fear of being single scale (Spielmann et al., 2013) | *It scares me to think that there might not be anyone out there for me* | X | X | | | X | X | | | X |
| Social media use | Self-constructed | Which social media platforms do you use most often? How often do you use X platform? |  |  | | |  |  | | |  |
| Forecasted changes | Self-constructed | *How satisfied do you think you will be with your close relationships after 3 months of your gender-affirming treatment?* | X |  | | |  |  | | |  |
| Implicit gender associations | Gender IAT task | *Behavioural measure* | X | X | | |  | X | | |  |
| **SOCIAL STIGMA** | | | | | | | | | | | |
| Social gender affirmation | Self-constructed | *People use my correct name when addressing me.* | X | X | | | X | X | | | X |
| Transgender minority stress | Subscales Vigilance, Foreshortened Futures, Vicarious frequency, Transitioning identity stress from the Transgender and nonbinary minority stress scales (Puckett et al., 2025) | *I worry that others will accidentally out me as trans.* | X | X | | | X | X | | | X |
| Sexual objectification | Interpersonal sexual objectification scale (ISOS) (Kozee et al., 2007) | *How often have you heard a rude, sexual remark made about your body?* | X | X | | | X | X | | | X |
| **GENDER CONGRUENCE** | | | | | | | | | | | |
| Gender congruence | Transgender Congruence Scale (TCS) (Kozee et al., 2012) | *My physical appearance adequately expresses my gender identity.* | X | X | | | X | X | | | X |
| Gender euphoria | Self-affirmation scale of the Gender euphoria scale (Blacklock et al., 2025) | *How strong has my euphoria been … when I see my face in the mirror* | X | X | | | X | X | | | X |
| Appearance satisfaction | Appearance scale of the Body Esteem Scale Adolescent Adults (BESAA)  (Mendelson et al., 2001) | *I like what I see when I look in the mirror.* | X | X | | | X | X | | | X |
| Body appreciation | Body Appreciation Scale-2  (Tylka & Wood-Barcalow, 2015) | *I feel good about my body.* | X | X | | | X | X | | | X |
| **PSYCHOSOCIAL FUNCTIONING** | | | | | | | | | | | |
| **The self** | | | | | | | | | | | |
| Self-efficacy | Pearlin Self-Mastery Scale  (Pearlin et al., 1981; Pearlin & Schooler, 1978) | *I can do just about anything I really set my mind to.* | X | X | | | X | X | | | X |
| Self-esteem | Rosenberg Self-Esteem Scale  (Rosenberg, 1965) | *On the whole, I am satisfied with myself.* | X | X | | | X | X | | | X |
| Self-consciousness | Body Surveillance subscale of the Objectified Body Consciousness Scale  (McKinley & Hyde, 1996) | *During the day‚ I think about how I look many times.* | X | X | | | X | X | | | X |
| Self-concept clarity | Campbell Self-concept Clarity Scale  (Campbell et al., 1996) | *In general, I have a clear sense of who I am and what I am.* | X | X | | | X | X | | | X |
| Authenthicity | State authenticity scale (Horton et al., 2025) | *I feel like myself.* | X | X | | | X | X | | | X |
| Self-control | Point Subtraction Aggression Paradigm  (Geniole et al., 2017) | *Behavioural measure* | X | X | | | X | X | | | X |
| **PSYCHOSOCIAL FUNCTIONING** | | | | | | | | | | | |
| **Interpersonal** | | | | | | | | | | | |
| Sexual satisfaction | Modified version of the Satisfaction with Sex Life Scale – Revised (Park & MacDonald, 2022) | *I am satisfied with my sex life.* | X | X | | | X | X | | | X |
| Interpersonal functioning | Interpersonal Competence Questionnaire-15  (Coroiu et al., 2015) | *Indicate how confident you feel in your ability to Introducing yourself to someone you might like to get to know/date.* | X | X | | | X | X | | | X |
| Fear of negative evaluation | Brief fear of negative evaluation scale  (Leary, 1983) | *I am afraid that others will not approve of me.* | X | X | | | X | X | | | X |
| Trust | Coin toss trust game (Long et al., 2012) | *Behavioural measure* | X | X | | | X | X | | | X |
| **PSYCHOSOCIAL FUNCTIONING** | | | | | | | | | | | |
| **Emotions** | | | | | | | | | | | |
| Life satisfaction | Satisfaction With Life Scale  (Diener et al., 1985) | *I am satisfied with my life.* | X | X | | | X | X | | | X |
| Depressive symptoms | Center for Epidemiologic Studies Depression Scale  (Radloff, 1977) | *I feel depressed.* | X | X | | | X | X | | | X |
| Anxiety symptoms | Self-Rating Anxiety Scale (Zung, 1971) | *I feel more nervous and anxious than usual.* | X | X | | | X | X | | | X |
| Emotion reactivity | Brief-Emotion Reactivity Scale (Veilleux et al., 2024) | *I tend to get very emotional very easily.* |  |  | | |  |  | | |  |
| Emotion regulation | Intrapersonal prompts for rumination, expressive suppression, calming and acceptance from the Emotion Regulation Strategy Scale  (Kneeland et al., 2024) | *When something happens that makes me feel an emotion that I want to change, I manage how I am feeling by trying to …*   - *Repeatedly analyze the situation that made me feel this way* (rumination) - *Not express how I feel* (expressive suppression)   *Take deep breaths* (calming)*Accept that I am feeling this emotion* (acceptance) | X | - X | | | X | X | | | X |
| Alexithymia | Perth Alexithymia Questionnaire-Short form  (Preece et al., 2023) | *When I’m feeling bad (feeling an unpleasant emotion), I can’t find the right words to describe those feelings.* | X | X | | | X | X | | | X |
| Emotional inhibition | Emotion-Face Stroop task  (Ros et al., 2023) | *Behavioural measure* | X | X | | | X | X | | | X |
| **SOCIAL HEALTH** | | | | | | | | | | | |
| **Global** | | | | | | | | | | | |
| Social well-being | Item from WHO-QOL BREF Questionnaire (Whoqol Group, 1998) | *How satisfied are you with your personal relationships?* | X | X | | | X | X | | | X |
| Social support | ENRICHD Social Support Inventory (Mitchell et al., 2003) | *Is there someone available to you whom you can count on to listen to you when you need to talk?* | X | X | | | X | X | | | X |
| Loneliness | 3-item version of the UCLA Loneliness Scale (Hughes et al., 2004) | *How often do you feel that you lack companionship?* | X | X | | | X | X | | | X |
| Relationship status satisfaction | Satisfaction with Relationship Status (ReSta) scale (Lehmann et al., 2015) | *In general, how satisfied are you with your current romantic relationship status?* | X | X | | | X | X | | | X |
| Social disconnection | Thwarted belonging subscale from the Interpersonal Needs Questionnaire (Van Orden et al., 2012) | *These days, other people care about me* | X | X | | | X | X | | | X |
| **Network Member Specific** | | | | | | | | | | | |
| Social network | Egocentric social network with sociogram in Network Canvas (Janulis et al., 2023) | *Measure assessing number, demographics, and relationship quality of network members* | X | X | | |  | X | | | X |

**References:**

Allison, C., Auyeung, B., & Baron-Cohen, S. (2012). Toward Brief “Red Flags” for Autism Screening: The Short Autism Spectrum Quotient and the Short Quantitative Checklist in 1,000 Cases and 3,000 Controls. *Journal of the American Academy of Child & Adolescent Psychiatry*, *51*(2), 202-212.e7. https://doi.org/10.1016/j.jaac.2011.11.003

Baber, K. M., & Tucker, C. J. (2006). The Social Roles Questionnaire: A new approach to measuring attitudes toward gender. *Sex Roles: A Journal of Research*, *54*(7-8), 459-467. https://doi.org/10.1007/s11199-006-9018-y

Blacklock, C. A., Tollit, M. A., Pace, C. C., Elphinstone, B., Zwickl, S., Cheung, A. S., Citron, K., Marsan, S., Zucker, N., Buzwell, S., & Pang, K. C. (2025). The Gender Euphoria Scale (GES): Development of a tool to measure gender euphoria. *International Journal of Transgender Health*, *0*(0), 1-12. https://doi.org/10.1080/26895269.2024.2447768

Campbell, J. D., Trapnell, P. D., Heine, S. J., Katz, I. M., Lavallee, L. F., & Lehman, D. R. (1996). Self-concept clarity: Measurement, personality correlates, and cultural boundaries. *Journal of Personality and Social Psychology*, *70*(1), 141-156. https://doi.org/10.1037/0022-3514.70.1.141

Coroiu, A., Meyer, A., Gomez-Garibello, C. A., Brähler, E., Hessel, A., & Körner, A. (2015). *Interpersonal Competence Questionnaire-15*. https://doi.org/10.1037/t48804-000

Crowne, D. P., & Marlowe, D. (1960). A new scale of social desirability independent of psychopathology. *Journal of Consulting Psychology*, *24*(4), 349-354. https://doi.org/10.1037/h0047358

Diener, E., Emmons, R. A., Larsen, R. J., & Griffin, S. (1985). The Satisfaction With Life Scale. *Journal of Personality Assessment*, *49*(1), 71-75. https://doi.org/10.1207/s15327752jpa4901_13

Fraley, R. C., Heffernan, M. E., Vicary, A. M., & Brumbaugh, C. C. (2011). The experiences in close relationships—Relationship Structures Questionnaire: A method for assessing attachment orientations across relationships. *Psychological Assessment*, *23*(3), 615-625. https://doi.org/10.1037/a0022898

Galupo, M. P., Lomash ,Edward, & and Mitchell, R. C. (2017). “All of My Lovers Fit Into This Scale”: Sexual Minority Individuals’ Responses to Two Novel Measures of Sexual Orientation. *Journal of Homosexuality*, *64*(2), 145-165. https://doi.org/10.1080/00918369.2016.1174027

Geniole, S. N., MacDonell, E. T., & McCormick, C. M. (2017). The Point Subtraction Aggression Paradigm as a laboratory tool for investigating the neuroendocrinology of aggression and competition. *Hormones and Behavior*, *92*, 103-116. https://doi.org/10.1016/j.yhbeh.2016.04.006

Horton, C. B. Jr., Bailey, E. R., & Iyengar, S. S. (2025). Pinning down state authenticity: Defining and validating a state authenticity measure. *Self and Identity*, *24*(1-2), 1-27. https://doi.org/10.1080/15298868.2024.2434750

Hughes, M. E., Waite, L. J., Hawkley, L. C., & Cacioppo, J. T. (2004). A Short Scale for Measuring Loneliness in Large Surveys. *Research on aging*, *26*(6), 655-672. https://doi.org/10.1177/0164027504268574

Hull, L., Mandy, W., Lai, M.-C., Baron-Cohen, S., Allison, C., Smith, P., & Petrides, K. V. (2019). Development and Validation of the Camouflaging Autistic Traits Questionnaire (CAT-Q). *Journal of Autism and Developmental Disorders*, *49*(3), 819-833. https://doi.org/10.1007/s10803-018-3792-6

Janulis, P., Phillips, G., Melville, J., Hogan, B., Banner, K., Mustanski, B., Oser, C. B., Tillson, M., Schneider, J., & Birkett, M. (2023). Network canvas: An open-source tool for capturing social and contact network data. *International Journal of Epidemiology*, *52*(4), 1286-1291. https://doi.org/10.1093/ije/dyad036

Kneeland, E. T., Hay, A., Curtiss, J., Hennessey, A., Vanderlind, W. M., Joormann, J., & Clark, M. S. (2024). The development of a novel scale to assess intra- and interpersonal emotion regulation strategies: The Emotion Regulation Strategy Scale (ERSS). *Emotion*, *24*(7), 1582-1599. https://doi.org/10.1037/emo0001375

Kozee, H. B., Tylka, T. L., Augustus-Horvath, C. L., & Denchik, A. (2007). Development and Psychometric Evaluation of the Interpersonal Sexual Objectification Scale. *Psychology of Women Quarterly*, *31*(2), 176-189. https://doi.org/10.1111/j.1471-6402.2007.00351.x

Kozee, H. B., Tylka, T. L., & Bauerband, L. A. (2012). Measuring Transgender Individuals’ Comfort With Gender Identity and Appearance: Development and Validation of the Transgender Congruence Scale. *Psychology of Women Quarterly*, *36*(2), 179-196. https://doi.org/10.1177/0361684312442161

Leach, C. W., van Zomeren, M., Zebel, S., Vliek, M. L. W., Pennekamp, S. F., Doosje, B., Ouwerkerk, J. W., & Spears, R. (2008). Group-level self-definition and self-investment: A hierarchical (multicomponent) model of in-group identification. *Journal of Personality and Social Psychology*, *95*(1), 144-165. https://doi.org/10.1037/0022-3514.95.1.144

Leary, M. R. (1983). A Brief Version of the Fear of Negative Evaluation Scale. *Personality and Social Psychology Bulletin*, *9*(3), 371-375. https://doi.org/10.1177/0146167283093007

Lehmann, V., Tuinman, M. A., Braeken, J., Vingerhoets, Ad. J. J. M., Sanderman, R., & Hagedoorn, M. (2015). Satisfaction with Relationship Status: Development of a New Scale and the Role in Predicting Well-Being. *Journal of Happiness Studies*, *16*(1), 169-184. https://doi.org/10.1007/s10902-014-9503-x

Long, Y., Jiang, X., & Zhou, X. (2012). To believe or not to believe: Trust choice modulates brain responses in outcome evaluation. *Neuroscience*, *200*, 50-58. https://doi.org/10.1016/j.neuroscience.2011.10.035

McKinley, N. M., & Hyde, J. S. (1996). The Objectified Body Consciousness Scale: Development and Validation. *Psychology of Women Quarterly*, *20*(2), 181-215. https://doi.org/10.1111/j.1471-6402.1996.tb00467.x

Mendelson, B. K., Mendelson, M. J., & White, D. R. (2001). Body-Esteem Scale for Adolescents and Adults. *Journal of Personality Assessment*, *76*(1), 90-106. https://doi.org/10.1207/S15327752JPA7601_6

Mitchell, P. H., Powell, L., Blumenthal, J., Norten, J., Ironson, G., Pitula, C. R., Froelicher, E. S., Czajkowski, S., Youngblood, M., Huber, M., & Berkman, L. F. (2003). A Short Social Support Measure for Patients Recovering From Myocardial Infarction: THE ENRICHD SOCIAL SUPPORT INVENTORY. *Journal of Cardiopulmonary Rehabilitation and Prevention*, *23*(6), 398.

Okechukwu, C. A., El Ayadi, A. M., Tamers, S. L., Sabbath, E. L., & Berkman, L. (2012). Household food insufficiency, financial strain, work-family spillover, and depressive symptoms in the working class: The Work, Family, and Health Network study. *American Journal of Public Health*, *102*(1), 126-133. https://doi.org/10.2105/AJPH.2011.300323

Park, Y., & MacDonald, G. (2022). Single and Partnered Individuals’ Sexual Satisfaction as a Function of Sexual Desire and Activities: Results Using a Sexual Satisfaction Scale Demonstrating Measurement Invariance Across Partnership Status. *Archives of Sexual Behavior*, *51*(1), 547-564. https://doi.org/10.1007/s10508-021-02153-y

Pearlin, L. I., Menaghan, E. G., Lieberman, M. A., & Mullan, J. T. (1981). The Stress Process. *Journal of Health and Social Behavior*, *22*(4), 337-356. https://doi.org/10.2307/2136676

Pearlin, L. I., & Schooler, C. (1978). The Structure of Coping. *Journal of Health and Social Behavior*, *19*(1), 2-21. https://doi.org/10.2307/2136319

Preece, D. A., Mehta, A., Petrova, K., Sikka, P., Bjureberg, J., Chen, W., Becerra, R., Allan, A., Robinson, K., & Gross, J. J. (2023). The Perth Alexithymia Questionnaire-Short Form (PAQ-S): A 6-item measure of alexithymia. *Journal of Affective Disorders*, *325*, 493-501. https://doi.org/10.1016/j.jad.2023.01.036

Puckett, J. A., DuBois, L. Z., Kimball, D., Huynh, K., & McCauley, H. L. (2025). Development and validation of novel measures of gender minority stress for transgender and nonbinary people. *International Journal of Transgender Health*, *0*(0), 1-19. https://doi.org/10.1080/26895269.2024.2447756

Radloff, L. S. (1977). The CES-D Scale: A Self-Report Depression Scale for Research in the General Population. *Applied Psychological Measurement*, *1*(3), 385-401. https://doi.org/10.1177/014662167700100306

Rammstedt, B., & John, O. P. (2007). Measuring personality in one minute or less: A 10-item short version of the Big Five Inventory in English and German. *Journal of Research in Personality*, *41*(1), 203-212. https://doi.org/10.1016/j.jrp.2006.02.001

Ros, L., Satorres, E., Fernández-Aguilar, L., Delhom, I., López-Torres, J., Latorre, J. M., & Melendez, J. C. (2023). Differential effects of faces and words in cognitive control in older adults with and without major depressive disorder: An emotional Stroop task study. *Applied Neuropsychology: Adult*, *30*(2), 239-248. https://doi.org/10.1080/23279095.2021.1927037

Rosenberg, M. (1965). *Society and the Adolescent Self-Image*. Princeton University Press. https://www.jstor.org/stable/j.ctt183pjjh

Spector, I. P., Carey ,Michael P., & and Steinberg, L. (1996). The sexual desire inventory: Development, factor structure, and evidence of reliability. *Journal of Sex & Marital Therapy*, *22*(3), 175-190. https://doi.org/10.1080/00926239608414655

Spielmann, S. S., MacDonald, G., Maxwell, J. A., Joel, S., Peragine, D., Muise, A., & Impett, E. A. (2013). Settling for less out of fear of being single. *Journal of Personality and Social Psychology*, *105*(6), 1049-1073. https://doi.org/10.1037/a0034628

Steiner, M., Macdougall, M., & Brown, E. (2003). The premenstrual symptoms screening tool (PSST) for clinicians. *Archives of Women’s Mental Health*, *6*(3), 203-209. https://doi.org/10.1007/s00737-003-0018-4

Tylka, T. L., & Wood-Barcalow, N. L. (2015). The Body Appreciation Scale-2: Item refinement and psychometric evaluation. *Body Image*, *12*, 53-67. https://doi.org/10.1016/j.bodyim.2014.09.006

Van Orden, K. A., Cukrowicz, K. C., Witte, T. K., & Joiner Jr., T. E. (2012). Thwarted belongingness and perceived burdensomeness: Construct validity and psychometric properties of the Interpersonal Needs Questionnaire. *Psychological Assessment*, *24*(1), 197-215. https://doi.org/10.1037/a0025358

Veilleux, J. C., Schreiber, R. E., Warner, E. A., & Brott, K. H. (2024). Development and validation of a brief version of the emotion reactivity scale: The B-ERS. *Current Psychology*, *43*(14), 12586-12600. https://doi.org/10.1007/s12144-023-05323-4

Whoqol Group, T. (1998). Development of the World Health Organization WHOQOL-BREF Quality of Life Assessment. *Psychological Medicine*, *28*(3), 551-558. https://doi.org/10.1017/S0033291798006667

Zung, W. W. K. (1971). *Self-Rating Anxiety Scale*. https://doi.org/10.1037/t04092-000
